# Comparative analyses of short‐ and long‐term outcomes between endoscopic submucosal dissection and endoscopic laryngo‐pharyngeal surgery for superficial pharyngeal carcinomas

**DOI:** 10.1002/deo2.70003

**Published:** 2024-09-12

**Authors:** Motomitsu Fukuhara, Yuji Urabe, Takeo Nakamura, Kazuki Ishibashi, Hirona Konishi, Junichi Mizuno, Takeshi Takasago, Hidenori Tanaka, Akiyoshi Tsuboi, Ken Yamashita, Yuichi Hiyama, Hidehiko Takigawa, Takahiro Kotachi, Ryo Yuge, Akira Ishikawa, Takayuki Taruya, Tsutomu Ueda, Sachio Takeno, Shiro Oka

**Affiliations:** ^1^ Department of Gastroenterology Graduate School of Biomedical and Health Sciences Hiroshima University Hiroshima Japan; ^2^ Gastrointestinal Endoscopy and Medicine Hiroshima University Hospital Hiroshima Japan; ^3^ Department of Clinical Research Center Hiroshima University Hospital Hiroshima Japan; ^4^ Department of Molecular Pathology Graduate School of Biomedical and Health Sciences Hiroshima University Hiroshima Japan; ^5^ Department of Otorhinolaryngology Head and Neck Surgery Graduate School of Biomedical Sciences Hiroshima University Hiroshima Japan

**Keywords:** endoscopic submucosal dissection, head and neck squamous cell carcinomas, otorhinolaryngological surgical procedures, squamous cell carcinoma of the head and neck, surgical procedures

## Abstract

**Objectives:**

Endoscopic treatment of superficial pharyngeal carcinomas includes endoscopic submucosal dissection (ESD; usually performed by endoscopists), and endoscopic laryngo‐pharyngeal surgery (ELPS; primarily performed by otolaryngologists). Few studies have compared the efficacy of the two techniques in treating superficial pharyngeal carcinomas. In this study, we compared the outcomes of these two techniques to determine the advantages.

**Methods:**

We retrospectively examined the short‐ and long‐term outcomes of 93 consecutive patients with superficial pharyngeal carcinoma who either underwent an ESD or ELPS between August 2008 and December 2021.

**Results:**

There were 35 lesions among 29 patients and 93 lesions among 71 patients in the ESD and ELPS groups, respectively. The ELPS group had a significantly shorter procedure time (121.2 ± 97.4 min vs. 54.7 ± 40.2 min, p<0.01), greater procedure speed (0.10 ± 0.06 min/min vs. 0.30 ± 0.23 min/min, p<0.01), and less laryngeal edema than that of the ESD group. There were no significant differences in the 3‐year overall, relapse‐free, or disease‐specific survival rates between the two groups. Intervention with ESD during ELPS was most commonly required when it was difficult to secure the visual field.

**Conclusions:**

There were no differences in batch resection rates or long‐term prognoses between the two groups; nevertheless, the ELPS group had a shorter treatment time and less laryngeal edema than the ESD group. However, the treatment of narrow areas, such as the esophageal inlet patch, is a technical limitation of ELPS; thus, ELPS should be combined with ESD techniques.

## INTRODUCTION

Laryngopharyngeal carcinoma is frequently in an advanced stage at detection and has a relatively poor prognosis.^1^ Advanced laryngopharyngeal carcinomas require either a total laryngectomy or chemoradiotherapy and considerably reduce laryngeal function and the quality of life.^2^ Early detection of tumors is crucial for increasing survival rates and minimizing functional impairments of swallowing and voice. Recently, the development and widespread use of diagnostic imaging techniques such as narrow‐band imaging have enabled the early detection of superficial pharyngeal carcinomas.^3^

Endoscopic resection is the first choice of treatment for superficial pharyngeal carcinomas (SPCs) owing to its excellent outcomes and low incidence of complications.^4^ Endoscopic resection for SPCs is more advantageous than endoscopic treatment for other organs because of the possibility of organ preservation and the high quality of life after surgery.^5^ Endoscopic treatment of SPCs has been performed using endoscopic mucosal resection (EMR) and endoscopic submucosal dissection (ESD), extrapolated from the guidelines for the endoscopic treatment of esophageal and gastric carcinomas. In 2004, Omori et al. developed endoscopic laryngo‐pharyngeal surgery (ELPS), in which the larynx is expanded using a curved rigid laryngoscope, forceps or electrocautery is inserted trans‐orally, and endoscopically assisted dissection of the subepithelial layer beneath the lesion is performed.^6^ Thus far, few studies have compared ELPS and ESD in the management of SPCs. Furthermore, the indications for these techniques are unclear. In this study, we compared the short‐ and long‐term outcomes of ESD and ELPS at our institution to determine the advantages of these two techniques.

## METHODS

### Ethical considerations

All enrolled participants were informed of the risks and benefits of ESD and ELPS and provided written informed consent for the use of their data. This study was approved by the Institutional Review Board of Hiroshima University Hospital (approval number: E2021‐2657) and was conducted in accordance with the principles of the Declaration of Helsinki.

### Study design and population

We retrospectively enrolled consecutive patients with SPCs who underwent ESD and ELPS at Hiroshima University Hospital between August 2008 and December 2021. Until September 2014, all patients were treated using ESD, and after October 2014, they were treated using ELPS. However, in cases where treatment with ELPS was technically impossible, such as when the ELPS forceps could not reach the patient, ESD was used from the beginning.

**FIGURE 1:**
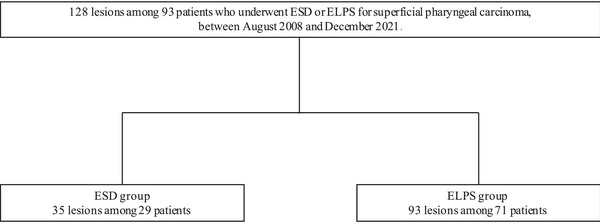
Study flowchart depicting the comparison of the short‐term outcomes between the patients who underwent ESD (the ESD group) and those who underwent ELPS (the ELPS group) for SPCs. ESD, endoscopic submucosal dissection; ELPS, endoscopic laryngo‐pharyngeal surgery; SPC, superficial pharyngeal carcinoma

The patients were divided into two groups: the ESD group (patients who underwent ESD alone) and the ELPS group (patients who underwent ELPS; Figure 1). We analyzed and compared the treatment outcomes between these two groups. The indications for concomitant ESD were analyzed for the patients in the ELPS group. Moreover, the long‐term prognosis was compared between the two groups to examine the 3‐year prognosis until the final date of December 2022.

### ELPS and ESD procedures

All procedures were performed under general anesthesia using a curved rigid laryngoscope (Nagashima Medical Instruments Company, Ltd.) to create a working space. A magnifying endoscope (GIF‐H260Z or GIF‐H290Z; Olympus Co.) was used for observation before treatment, and a single‐channel upper gastrointestinal endoscope with a water‐jet system (GIF‐Q260 J; Olympus) was used during treatment. The extent of the lesion was determined by the findings of the narrow‐band imaging endoscopic examination and iodine staining.

For ESD, a solution consisting of glycerin (10%) and indigo carmine (0.005%) was injected into the submucosal layer for lifting. Next, the lesion was marked using DualKnife or DualKnife J (Olympus Medical Systems), and an incision and dissection were performed. During this procedure, gastroenterologists performed the resection with the assistance of an otolaryngologist. We used clip‐line traction in all cases.

For ELPS, after the submucosal injection and lifting, the lesion was resected manually using a curved needle knife (Olympus Optical Co.) and curved or Steiner's forceps. The resection was performed by otolaryngologists, with the assistance of gastrointestinal endoscopists. Traction was performed with forceps in all patients. In cases where the otorhinolaryngologist determined that resection or dissection with ELPS was difficult, ESD techniques were used in combination. The decision to return to the ELPS was reached after mutual consultation between the gastrointestinal endoscopist and otolaryngologist. These patients were treated using ELPS to the greatest extent possible. A needle scalpel was used when the ESD technique was performed during ELPS. If pre‐operative assessment determined that ELPS would not be possible, such as in cases where the patient had a narrow oral cavity, the tumor was treated using only the ESD technique. ESG100 electrosurgical generators (Olympus) were used until 2009; thereafter, VIO300D generators (ERBE Elektromedizin GmbH) were used. The two gastrointestinal endoscopists who performed the treatments were certified by the Japan Gastroenterological Endoscopy Society and had >10 years of medical experience, involving >100 ESD cases. The otolaryngologist is a well‐trained head and neck surgeon, and the two endoscopists all had > 10 years of case experience. We followed up with an endoscopy and cervical echocardiography every 6 months as a post‐treatment follow‐up.

### Definitions

Definitions are presented in detail in Supporting Information 1.

### Pathological examinations

Pathological examinations are presented in detail in Supporting Information 1.

### Evaluation

The primary outcomes were the complete resection rate and prognosis. The secondary outcomes were the procedure time, histopathologic findings, and adverse event occurrence. Moreover, we investigated the clinical characteristics of lesions that required resection with ESD techniques in patients in the ELPS group.

### Statistical analyses

Categorical variables, expressed as percentages, were statistically compared using the chi‐square and Fisher's exact tests. Continuous variables, expressed as means ± standard deviations or as medians (ranges), were compared using Student's t‐test. The Kaplan–Meier method was used to calculate survival rates. The log‐rank test was used to compare survival curves in the univariate analysis. Statistical analyses were performed using JMP version 15 (SAS Institute Inc.). Statistical significance was set at *p* < 0.05.

## RESULTS

### Participant and tumor characteristics

We enrolled 93 patients (128 SPCs) who underwent ESD or ELPS (Figure 1). The characteristics of the patients and tumors are presented in Table 1. The ESD and ELPS groups included 29 (35 lesions) and 71 patients (93 lesions), respectively. The characteristics of the patients and tumors for the ESD and ELPS groups are shown in Table 2. The observation period was significantly longer in the ESD group than in the ELPS group (87.6 ± 51.1 months vs. 37.7 ± 23.8 months, *p* < 0.01).

**TABLE 1:** Overall patient and tumor characteristics.

Variables	
Age, years, mean ± SD	68.2 ± 9.1
Sex	
Male, *n* (%)	119 (93.0)
Female, *n* (%)	009 (07.0)
JCOG‐PS	
0	109 (85.2)
1	017 (13.2)
2	002 (01.6)
Observation period (months), mean ± SD	51.4 ± 40.1
Location	
Oropharynx, *n* (%)	028 (21.9)
Posterior wall	015 (11.8)
Lateral wall	004 (03.1)
Anterior wall	009 (07.0)
Hypopharynx, *n* (%)	100 (78.1)
Pyriform sinus	082 (64.0)
Postcricoid	001 (00.8)
Posterior wall	013 (10.2)
Esophageal inlet	004 (03.1)
Tumor size, mm, mean ± SD	22.9 ± 11.4
Macroscopic type	
0‐I, *n* (%)	017 (13.3)
0‐IIa, *n* (%)	057 (44.5)
0‐IIb, *n* (%)	037 (28.9)
0‐IIc, *n* (%)	017 (13.3)
JES‐SCC	
Type B1 vessels, *n* (%)	097 (75.8)
Type B2 vessels, *n* (%)	021 (16.4)
Type B3 vessels, *n* (%)	010 (07.8)
Procedure	
ESD	035 (27.3)
ELPS	093 (72.7)

There were 128 lesions in 93 patients who underwent ESD/ELPS for superficial pharynx squamous cell carcinoma between August 2008 and December 2021.

Abbreviations: ELPS, endoscopic laryngo‐pharyngeal surgery; ESD, endoscopic submucosal dissection; JCOG‐PS, Japan Clinical Oncology Group Performance Status; JES, Japan Esophageal Society; SCC, squamous cell carcinoma; SD, standard deviation.

**TABLE 2:** Patient and tumor characteristics of endoscopic submucosal dissection/endoscopic laryngo‐pharyngeal surgery.

	ESD group	ELPS group	
Variables	*n* = 35	*n* = 93	*p*‐value
Age, years, mean ± SD	66.9 ± 10.4	68.7 ± 8.6	0.3364
Sex			
Male, *n* (%)	33 (94.3)	86 (92.5)	1
Female, *n* (%)	02 (05.7)	07 (07.5)	
JCOG‐PS			
0	29 (82.9)	80 (86.0)	0.7807
1/ 2	06 (17.1)	11 (14.0)	
Observation period (months), mean ± SD	87.6 ± 51.1	37.7 ± 23.8	<0.01
Location			
Oropharynx, *n* (%)	07 (20.0)	21 (22.6)	0.8153
Posterior wall	05 (14.3)	10 (10.8)	0.5522
Lateral wall	00 (00.0)	04 (04.3)	0.5743
Anterior wall	02 (05.7)	07 (07.5)	1.0000
Hypopharynx, *n* (%)	28 (80.0)	72 (77.4)	0.8153
Pyriform sinus	21 (60.0)	61 (65.6)	0.6798
Postcricoid	00 (00.0)	01 (01.1)	1.0000
Posterior wall	04 (11.4)	09 (09.7)	0.7502
Esophageal inlet	03 (08.6)	01 (01.1)	0.0620
Tumor size, mean ± SD	21.9 ± 11.1	23.3 ± 11.5	0.5363
Macroscopic type			
0‐I, *n* (%)	03 (08.6)	14 (15.1)	0.3982
0‐IIa, *n* (%)	18 (51.4)	39 (41.9)	0.3364
0‐IIb, *n* (%)	12 (34.3)	25 (26.9)	0.4149
0‐IIc, *n* (%)	02 (05.7)	15 (16.1)	0.1520
JES‐SCC			
Type B1 vessels, *n* (%)	30 (85.7)	67 (72.0)	0.1638
Type B2 vessels, *n* (%)	05 (14.3)	16 (17.2)	0.7940
Type B3 vessels, *n* (%)	00 (00.0)	10 (10.8)	0.0608

We categorized all 128 lesions in 93 patients into the ESD and ELPS groups. The ESD group included 35 lesions in 29 patients. The ELPS group included 93 lesions in 71 patients.

Abbreviations: ELPS, endoscopic laryngo‐pharyngeal surgery; ESD, endoscopic submucosal dissection; JCOG‐PS, Japan Clinical Oncology Group Performance Status; JES, Japan Esophageal Society; SCC, squamous cell carcinoma; SD, standard deviation.

### Short‐term treatment outcomes

The short‐term treatment outcomes are presented in Table 3. The ELPS group had a significantly shorter procedure time (121.2 ± 97.4 min vs. 54.7 ± 40.2 min, *p* < 0.01) and greater procedure speed (0.10 ± 0.06 min/min vs. 0.30 ± 0.23 min/min, *p* < 0.01) than that of the ESD group. Laryngeal edema incidence was significantly lower in the ELPS group than in the ESD group (85.7% [30/35] vs. 54.8% [51/93], *p* < 0.01).

**TABLE 3:** Short‐term treatment outcomes of the endoscopic submucosal dissection and endoscopic laryngo‐pharyngeal surgery groups.

	ESD group	ELPS group	
Variables	*n* = 35	*n* = 93	*p*‐value
En bloc resection, *n* (%)	34 (97.1)	92 (98.9)	0.4737
Complete en bloc resection, *n* (%)	32 (91.4)	90 (96.8)	0.3443
Resection size, mean ± SD	36.6 ± 13.7	38.0 ± 13.3	0.6003
Procedure time (min), mean ± SD	121.2 ± 97.4	54.7 ± 40.2	<0.01
Procedure speed (cm^2^ /min), mean ± SD	0.10 ± 0.06	0.30 ± 0.23	<0.01
Depth of tumor invasion			
CIS, *n* (%)	21 (60.0)	52 (55.9)	0.6947
SEP, *n* (%)	14 (40.0)	41 (44.1)	
Tumor thickness < 1000 µm, *n* (%)	12 (85.7)	23 (56.1)	0.0587
Tumor thickness ≥ 1000 µm, *n* (%)	02 (14.3)	18 (43.9)	
Vascular invasion, *n* (%)	01 (2.9)	08 (8.6)	0.4426
Lymphatic invasion, *n* (%)	00 (.0)	04 (4.3)	0.5743
Adverse events			
Laryngeal edema	30 (85.7)	51 (54.8)	<0.01
Delayed bleeding, *n* (%)	01 (2.9)	02 (2.2)	1
Dysphagia, *n* (%)	11 (31.4)	17 (18.3)	0.1179

We categorized all 128 lesions among the 93 patients into the ESD and ELPS groups. The ESD group includes 35 lesions among 29 patients. The ELPS group includes 93 lesions among 71 patients.

Abbreviations: CIS, carcinoma in situ; ELPS, endoscopic laryngo‐pharyngeal surgery; ESD, endoscopic submucosal resection; SD, standard deviation; SEP, subepithelial propria.

* Statistical significance was set at *p* < 0.05.

### Combination treatment with ELPS and ESD

We investigated the indications that led to the use of the ESD technique (35/101, 35%). The indications for combination treatment with ELPS and ESD are shown in Table 4. The most common indication (22 lesions; 62.9%) was “difficulty in securing the visual field due to the location of the lesion adjacent to the esophageal inlet patch.” This was followed by the indication “incision along the upper aspect of the glottis” (four lesions; 11.1%).

**TABLE 4:** Indications for concomitant treatment using both the endoscopic submucosal dissection and endoscopic laryngo‐pharyngeal surgery techniques.

Variables	*n* = 35
Difficulty in securing the visual field due to the location of the lesion adjacent to the esophageal inlet patch, *n* (%)	22 (62.9)
An incision along the upper aspect of the glottis, *n* (%)	04 (11.1)
Difficulty in securing the visual field due to the location of the lesion at the tongue root, *n* (%)	03 (8.6)
Narrow surgical field, *n* (%)	03 (8.6)
Difficulty in securing the visual field due to the location of the lesion at the pharyngeal–jejunal anastomosis, *n* (%)	01 (2.9)
An incision along the nasopharyngeal aspect, *n* (%)	01 (2.9)
Initial introduction of ELPS, *n* (%), *n* (%)	01 (2.9)

The indications for the ESD technique for 35 lesions are shown; it was either chosen during treatment, when the ELPS technique proved challenging, or preoperatively when the ESD technique was expected to be less challenging than the ELPS technique.

Abbreviations: ELPS, endoscopic laryngo‐pharyngeal surgery; ESD, endoscopic submucosal resection.

Additionally, 27 lesions adjacent to the esophageal inlet were classified into ESD‐alone (eight lesions) and ELPS+ESD (19 lesions) groups. Table 5 presents the short‐term outcomes for lesions near the esophageal inlet in the ESD‐alone and ELPS+ESD groups. No difference was found in the complete en bloc resection rate between the two groups. Additionally, no difference was found in the resection size; however, the ELPS+ESD group had shorter procedure times (140 ± 24.5 vs. 75.4 ± 50.9 min, *p* < 0.01) and faster operative speeds (0.08 ± 0.02 vs. 0.26 ± 0.15 cm^2^/min, *p* < 0.01) than did the ESD‐alone group. The rate of laryngeal edema did not differ between the two groups.

**TABLE 5:** Comparison of outcomes of the endoscopic submucosal dissection and endoscopic laryngo‐pharyngeal surgery + endoscopic submucosal dissection groups for lesions near the esophageal inlet.

	ESD‐group	ELPS+ESD group	
Variables	*n* = 8	*n* = 19	*p*‐value
En bloc resection, *n* (%)	08 (100)	19 (100)	1
Complete en bloc resection, *n* (%)	07 (87.5)	19 (100)	0.2963
Resection size, mean ± SD	40.6 ± 7.7	41.3 ± 15.2	0.7410
Procedure time (min), mean ± SD	140 ± 24.5	75.4 ± 50.9	<0.01
Procedure speed (cm^2^/min), mean ± SD	0.08 ± 0.02	0.26 ± 0.15	<0.01
Depth of tumor invasion			
CIS, *n* (%)	04 (50.0)	12 (63.2)	0.6754
SEP, *n* (%)	04 (50.0)	07 (36.8)	
Tumor thickness < 1000 µm, *n* (%)	03 (75.0)	4 (57.1)	1.0000
Tumor thickness ≥ 1000 µm), *n* (%)	01 (25.0)	3 (42.9)	
Vascular invasion, *n* (%)	00 (00.0)	00 (00.0)	0
Lymphatic invasion, *n* (%)	00 (00.0)	01 (05.3)	1.0000
Adverse events			
Laryngeal edema	06 (75.0)	16 (84.2)	0.6159
Delayed bleeding, *n* (%)	00 (00.0)	00 (00.0)	0
Dysphagia *n* (%)	03 (37.5)	05 (26.3)	0.6578
Local recurrence, *n* (%)	01 (12.5)	00 (00.0)	0.2963

We compared the outcomes of the ESD‐alone (eight cases) and ELPS+ESD (19 cases) groups for lesions near the esophageal inlet.

Abbreviations: CIS, carcinoma in situ; ELPS, endoscopic laryngo‐pharyngeal surgery; ESD, endoscopic submucosal resection; SD, standard deviation; SEP, subepithelial propria.

### Long‐term treatment outcomes

**FIGURE 2:**
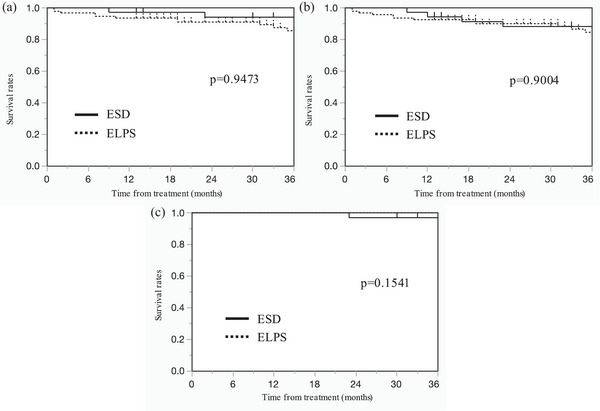
(a) The 3‐year overall survival (OS) curves of the ESD and ELPS groups. (b) The 3‐year RFS curves of the ESD and ELPS groups. (c) The 3‐year DSS curves of the ESD and ELPS groups. OS, overall survival; ESD, endoscopic submucosal dissection; ELPS, endoscopic laryngo‐pharyngeal surgery; RFS, relapse‐free survival; DSS, disease‐specific survival.

The long‐term prognoses for ESD and ELPS are summarized in Table 6 and Figure 2. The observation period was longer in the ESD group than in the ELPS group (87.6 ± 51.1 months vs. 37.7 ± 23.8 months, *p* < 0.01). Furthermore, there was a significant difference in other disease mortality rates between the two groups (40.0% [14/35] vs. 18.3% [17/93], *p* < 0.01), and only one cause‐specific death occurred in the ESD group. The 3‐year overall survival (OS) rates for the ESD and ELPS groups were 94.1% and 85.5%, respectively. The 3‐year relapse‐free survival rates for the ESD and ELPS groups were 88.2% and 84.5%, respectively. Moreover, the 3‐year disease‐specific survival rates for the ESD and ELPS groups were 96.9% and 100%, respectively.

**TABLE 6:** Long‐term treatment outcomes of the endoscopic submucosal dissection and endoscopic laryngo‐pharyngeal surgery groups.

	ESD group	ELPS group	
Variables	*n* = 35	*n* = 93	*p*‐value
Observation period (months), mean ± SD	87.6 ± 51.1	37.7 ± 23.8	<0.01
Recurrences	02 (5.8)	05 (5.4)	1
Local recurrences, *n* (%)	01 (2.9)	03 (3.2)	1
Lymph node metastases, *n* (%)	01 (2.9)	02 (2.2)	1
Cause of death	15 (42.9)	17 (18.3)	<0.01
Cause‐specific death, *n* (%)	01 (2.9)	00 (.0)	0.2734
Death of other diseases, *n* (%)	14 (40.0)	17 (18.3)	0.0192
3‐year OS	094.1	085.5	0.9473
3‐year RFS	088.2	084.5	0.9004
3‐year DSS	096.9	100.0	0.1541

We categorized all 128 lesions among the 93 patients into the ESD and ELPS groups. The ESD group includes 35 lesions among 29 patients. The ELPS group includes 93 lesions among 71 patients.

Abbreviations: DSS, disease‐specific survival; ELPS, endoscopic laryngo‐pharyngeal surgery; ESD, endoscopic submucosal resection; OS, overall survival; RFS, relapse‐free survival; SD, standard deviation.

* Statistical significance was set at *p* < 0.05.

## DISCUSSION

Our study showed that there were no statistically significant differences in the complete en bloc resection rates and long‐term prognoses between the ELPS and ESD groups; however, the ELPS group had a shorter treatment time and lower incidence of laryngeal edema than did the ESD group. The ESD technique used in combination with the ELPS technique was beneficial in cases where the lesions were adjacent to the esophageal inlet patch.

Endoscopic treatments such as EMR, ESD, and ELPS are considered safe and effective in the treatment of SPCs. When compared to using EMR in the treatment of SPCs, ESD resulted in a higher percentage of en bloc and microscopically margin‐negative resections in which no gross or microscopic tumor remained in the primary tumor bed (R0 resections); however, the treatment time was longer.^7^, ^8^, ^9^ En bloc resection with a snare in the complex anatomy of the pharyngeal region is challenging, and the EMR en bloc resection rate is low. ESD has a higher en bloc resection rate than EMR; nevertheless, ESD is technically more challenging and requires a longer operative time due to its single‐arm endoscopy procedure.^10^, ^11^, ^12^

Nakayama et al. reported that ELPS had the same resection rate but a shorter treatment time than that of ESD; their findings were similar to those of this study.^8^ The concept of ELPS is similar to that of ESD in that the tumor is resected en bloc, subsequent to submucosal injection; however, it differs from ESD in that the resection technique is performed by a surgeon using both hands.^6^ This allows the surgeon to manipulate the trans‐orally inserted forceps, grasp the lesion using the electrosurgical needle knife, and make a fine incision through the mucosa, while a clear view of the monitor is provided for the endoscopist. Resection with traction is essential because of the three‐dimensional anatomical features of the pharynx. ELPS allows for the use of both hands to adjust the direction of traction for resection, leading to a shorter treatment time.^1^ Additionally, ELPS allows the depth of dissection to be adjusted, according to the endoscopically predicted depth of the tumor.^13^ The subepithelial injection contributes to the preservation of deep structures. Thus, in lesions that are clearly diagnosable as intraepithelial lesions, minimizing resections can reduce unnecessary damage to the surrounding tissues and scar formation.^14^ Additionally, B2 and B3 vessels of the JES classification and 0‐IIa and 0‐I of the macroscopic types are highly likely to be invasive carcinomas of the subepithelial propria (SEP). Moreover, an invasive distance of 1000 µm is an independent risk factor for cervical lymph node metastases.^15^, ^16^, ^17^ Therefore, lesions with suspected SEP invasion should be dissected just above the muscle layer during ELPS for an en‐bloc resection. Regarding adverse events, there were no statistically significant differences in delayed bleeding or dysphagia between the two groups; however, laryngeal edema was less common in the ELPS group. Laryngeal edema is more likely to occur when the perilaryngeal local anesthetic injection volume is high, or with extended treatment time. The most effective method for reducing the amount of perilaryngeal local injection is exfoliation while applying traction, which allows for shorter treatment times.^18^ ESD allows traction in only one direction with clip line traction, whereas ELPS allows traction in multiple directions with forceps. Therefore, ELPS may have reduced local injection volume, shortened treatment time, and reduced laryngeal edema, compared with ESD. Previous radiation therapy for head and neck cancer is an independent risk factor for laryngeal edema after endoscopic treatment of SPC.^19^ Therefore, in cases with risk factors for laryngeal edema, such as a history of post‐radiotherapy, it may be preferable to choose ELPS, which has a shorter treatment time and a lower local injection volume.

The ELPS technique can generally be performed in the treatment of most parts of the body; however, it does not suffice in narrow and difficult‐to‐treat areas, such as the esophageal inlet patch, supraglottis, and epiglottis valleys. Furthermore, narrow and complex structures, such as the pyriform sinus and tongue root, are risk factors for incomplete endoscopic en bloc resection.^20^ ESD resection involves the use of a single instrument, such as the ESD knife or forceps, which can be applied to narrow areas, including adjacent to the esophageal inlet patch.^21^ In our study, using the ESD technique in such areas made it easier to approach the narrow cavities directly with the scope, as well as to check and resect the edges of the lesion. Furthermore, R0 resections were made possible.^21^ Therefore, ESD can potentially be used in such cases. Hybrid treatment using the ELPS and ESD techniques is useful in cases of hypopharyngeal carcinomas that partially invade the esophageal inlet patch.^1^, ^12^, ^22^, ^23^ In this study, we identified the need for ESD techniques in addition to ELPS in the treatment of the esophageal inlet patch, larynx, and glottis valley. We believe that the treatment of the sites identified in this study is a technical limitation of ELPS and that the addition of ESD techniques is necessary. The major advantage of endoscopic treatment with a curved rigid laryngoscope is that it provides a revolutionary wide view from the posterior cricoid to the cervical inlet patch, which is difficult to achieve using conventional methods.^22^ The use of a laryngoscope also enabled the creation of a good field‐of‐view of the supraglottis, epiglottis, and tongue root, making it possible for ELPS to approach areas that are difficult to resect en bloc with ESD.^13^ Owing to the varying shapes and sizes of the pharynx among patients, there are individualized differences in the areas where the instruments may interfere. Depending on the clinical situation, the use of either or both techniques is important.

In this study, the long‐term prognoses of the ESD and ELPS groups were comparable. There was no significant difference in the 3‐year survival rates between the two groups, and the survival subsequent to the use of the ELPS technique was comparable to that of ESD, as in previous reports.^1^, ^24^ Additionally, there were cases of local recurrences and lymph node metastases; however, only one case of primary mortality occurred in the ESD group. In this study, death from other causes was predominantly observed in the ESD group compared to the ELPS group, but this may be due to the longer observation period. There was no significant difference in the 3‐year DSS rates between the two groups, and the rate for the ELPS group was 100%, similar to that in previous reports.^1^, ^10^, ^24^, ^25^ In this study, salvage surgery or additional treatments were performed in most cases of local recurrences or lymph node metastases, and mortality from the primary disease was avoided. Pathologically positive margins are frequently caused by contusions from grasping or burning during excision; thus, local recurrence is not truly common in SPCs. Therefore, positive margins do not affect OS. However, in cases where there is a risk of local recurrence or lymph node metastases, follow‐up with periodic endoscopy, cervical ultrasonography, and computed tomography is essential, as salvage surgery or additional treatment should be performed as soon as possible.^8^

This study had certain limitations. This was a single‐center, retrospective study with a small sample size. Therefore, a large‐scale, multicenter, prospective study is required. The treatment choice after October 2014 may be a limitation as it was based on the endoscopist's and otolaryngologist's discretion. A significant difference was found in duration between the groups, and the endoscopists who performed pharyngeal ESD may have also performed ELPS, resulting in a difference in the technique proficiency. Furthermore, the amount of local injection has not been evaluated, although the laryngeal edema may be affected by the injection during the procedure.

In conclusion, no differences were found in the en bloc resection rates or long‐term outcomes between the ESD and ELPS groups. The ELPS group had a shorter treatment time and lower incidence of laryngeal edema than the ESD group. However, the treatment of narrow areas, such as the esophageal inlet patch, is a technical limitation of ELPS. Thus, ELPS should be combined with ESD techniques.

## CONFLICT OF INTEREST STATEMENT

None.

## ETHICS STATEMENT

All enrolled participants were informed of the risks and benefits of ESD and ELPS and provided written informed consent for the use of their data. This study was approved by the Institutional Review Board of Hiroshima University Hospital (approval number: E2021‐2657) and was conducted in accordance with the principles of the Declaration of Helsinki.

## Supporting information

Supporting_Information_1 Details of definitions and pathological examinations.

## Data Availability

The data that support the findings of this study are available from the corresponding author upon reasonable request.
