# Supplementary material for: Comparative analyses of short‐ and long‐term outcomes between endoscopic submucosal dissection and endoscopic laryngo‐pharyngeal surgery for superficial pharyngeal carcinomas
Source: DEN Open. 2024 Sep 12;5(1):e70003. doi: 10.1002/deo2.70003 (PMC11391100; doi:10.1002/deo2.70003)
Supplement: Supplementary file 1 — Supporting_Information_1 Details of definitions and pathological examinations. [file DEO2-5-e70003-s001.docx]

Doc. S1

***Definitions***

Macroscopic lesion types were classified as 0-I, 0-IIa, 0-IIb, 0-IIc, and 0-III, based on the Japanese Classification of Esophageal Cancer (11th edition).^1^ Magnifying endoscopy with narrow-band imaging microvascular patterns were classified according to the Japan Esophageal Society classification,^1^ which categorizes vessels as type A, B1, B2, and B3. Laryngeal edema was defined by the need for extubation of the tracheal tube 1–2 days following intubation. Dysphagia was defined as stage 2 or higher on the functional outcome swallowing scale.^2^ The procedure time was defined as the time from the end of marking to the end of excision.

**References**

1 Japan Esophageal Society. Japanese classification of esophageal cancer, 11th Edition: part1. *Esophagus*. 2017; **14**: 1–36.

2 Salassa JR. A functional outcome swallowing scale for staging oropharyngeal dysphagia. *Dig Dis* 1999; **17**: 230–4.

***Pathological examinations***

All specimens were pinned onto polystyrene receivers to facilitate histopathological sectioning, prior to immediate fixation in 10% buffered formalin solution. The specimens were cut into 2-mm-wide slices on the day after fixation. The slices were embedded in paraffin, cut into 3-μm-thick sections, stained with hematoxylin and eosin, and microscopically examined to ascertain the histological tumor type.
